# Aneurysmal Superior Mesenteric Artery Syndrome

**DOI:** 10.7759/cureus.24761

**Published:** 2022-05-05

**Authors:** Jun Kamei, Akira Kuriyama

**Affiliations:** 1 Emergency and Critical Care Center, Kurashiki Central Hospital, Kurashiki, JPN

**Keywords:** spinal cord ischemia, acute aortic dissection, case report, superior mesentery artery syndrome, abdominal aortic aneurysm

## Abstract

A 67-year-old man developed an abdominal aortic aneurysm (AAA) and Stanford type B acute aortic dissection. He received liberal antihypertensives for complicated spinal cord ischemia and, subsequently, experienced loss of appetite, followed by vomiting without abdominal pain. Computed tomography revealed AAA expansion and compression of the duodenum between the superior mesenteric artery (SMA) and AAA. He was diagnosed with aneurysmal SMA syndrome. Gastrointestinal symptoms in patients with an AAA can be a warning sign of SMA syndrome due to aortic aneurysm expansion, which can progress within a short time with accompanying acute aortic dissection.

## Introduction

Superior mesenteric artery (SMA) syndrome is a rare disease defined by the compression of the duodenum between the SMA and abdominal aorta [1]. SMA syndrome is commonly caused by weight loss and a decreased aortomesenteric angle by intra-abdominal fat reduction [2]. Cases of SMA syndrome associated with abdominal aortic aneurysm (AAA), or aneurysmal SMA syndrome, have been reported [3-5]. In previous reports, patients were admitted after they had developed gastrointestinal symptoms due to a gradually expanding aneurysm. We report a case of aneurysmal SMA syndrome complicated and progressed by acute aortic dissection.

## Case presentation

**Figure 1:**
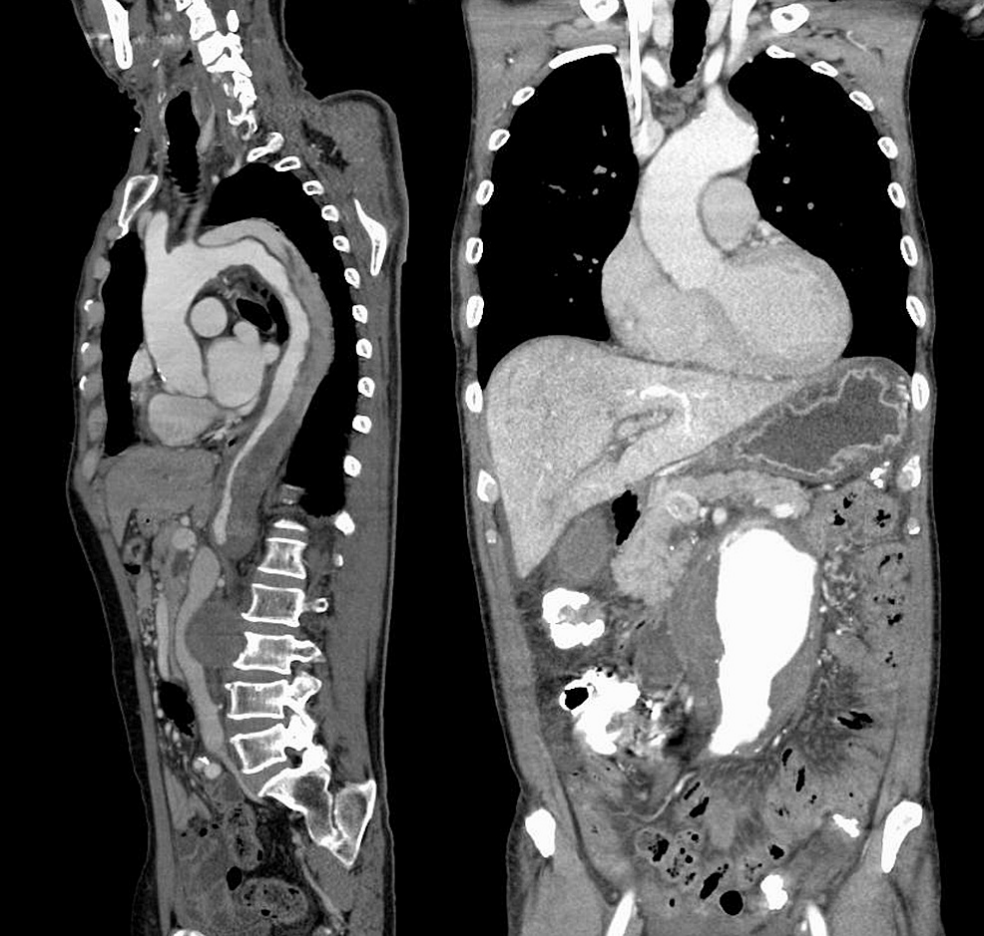
Chest and abdominal CT. Chest and abdominal contrast-enhanced CT show a Stanford type B aortic dissection and abdominal aortic aneurysm. CT: computed tomography

**Figure 2:**
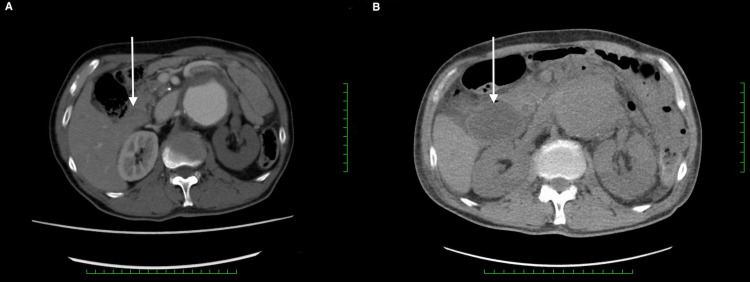
Abdominal CT on admission (A) and day six (B). The duodenum (white arrow) was not dilated on admission (A) but dilated on day six (B). CT: computed tomography

**Figure 3:**
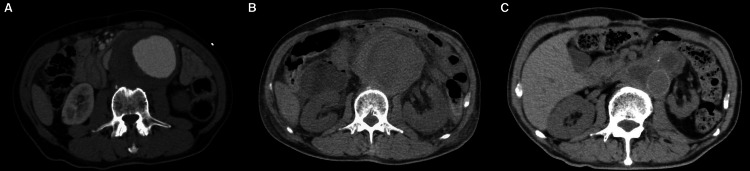
Abdominal CT on admission (A), day six (B), and postoperatively (C). Abdominal CT shows expansion of the abdominal aortic aneurysm from admission (A) to day six (B). Postoperative abdominal CT shows graft-replaced abdominal aorta and the decompressed duodenum (C). CT: computed tomography

**Figure 4:**
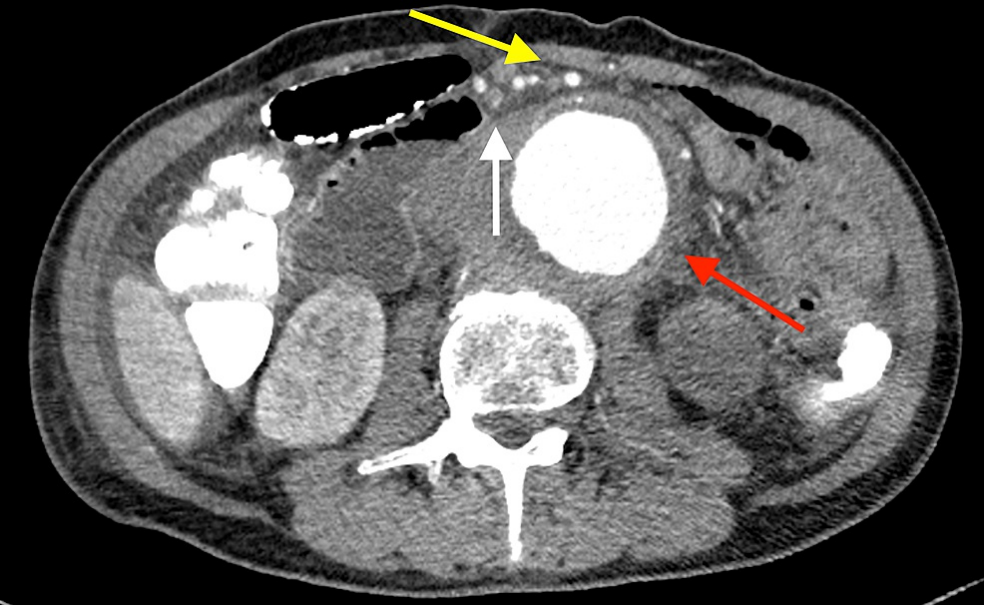
Abdominal CT. The duodenum (white arrow) is compressed between the superior mesenteric artery (yellow arrow) and abdominal aortic aneurysm (red arrow). CT: computed tomography

A 67-year-old man, with no significant past medical history, presented to our emergency department with severe chest and back pain. He awakened early in the morning with sudden onset of pain in the left chest, which gradually radiated to his back and abdomen. Computed tomography (CT) findings revealed the presence of an AAA and thrombosed dissected lumen from the descending aorta to above the abdominal aorta (Figure 1). The patient was diagnosed with Stanford type B acute aortic dissection because of the sudden onset of symptoms. There was no gastrointestinal tract dilatation or gastrointestinal obstruction (Figure 2A). He had bilateral lower extremity weakness of manual muscle testing (MMT) 3, with flaccid paralysis and hypoesthesia in the left leg. He had a sensory disorder below the sixth level of the thoracic spinal cord. He was clinically diagnosed with spinal cord ischemia due to malperfusion of aortic dissection. He received liberal antihypertensives for complicated spinal cord ischemia to achieve a systolic blood pressure below 130 mmHg. On day six, his appetite reduced, followed by vomiting without abdominal pain. Abdominal examination revealed normal bowel sounds, no distension, and no tenderness. CT revealed that the maximum diameter of AAA had increased from 7.4 to 8.0 cm (Figures 3A, 3B) and the duodenum was dilated (Figure 2B). The third portion was compressed between the SMA and AAA (Figure 4). On day eight, although the nasogastric tube could decompress the gastric and duodenal dilatation, abdominal radiography showed retention of contrast media in the stomach. The patient was diagnosed with SMA syndrome. Gastrointestinal endoscopy posed a risk of aneurysm rupture. Due to the large AAA having a risk of rupture, and the possibility of malnutrition developing due to duodenal obstruction unless the SMA syndrome resolved, we determined that the AAA warranted surgery. On day 17, a thoracoabdominal aortic replacement was performed. The portion from the descending aorta to the site above the aortic bifurcation was replaced. This portion had the segment of aortic dissection and aneurysm. The postoperative course was uneventful. The patient was able to intake foods orally. The lower extremity paralysis of MMT 3 persisted. One month after the surgery, he was transferred to the rehabilitation hospital. The postoperative CT showed that the duodenum had been decompressed (Figure 3C).

## Discussion

SMA syndrome is defined as the compression of the third portion of the duodenum between the SMA and abdominal aorta. The main pathophysiology involves a decrease in the aortomesenteric angle caused by weight loss and mesenteric fat pad reduction between the SMA and abdominal aorta [1,2]. In several reports, SMA syndrome, associated with AAA, developed when the duodenum was compressed between the SMA and expanded abdominal aorta [3-5].

The aortic dissection likely contributed to the AAA expansion because of two possible reasons. First, spinal cord ischemia had already developed due to the complications of Stanford type B acute aortic dissection upon admission. The blood pressure was controlled at higher targets than the standard therapy (lowering systolic blood pressure to 100-120 mmHg) for aortic dissection and AAA to maintain spinal perfusion pressure [6]. This liberal antihypertensive therapy might have caused an increase in blood pressure and, subsequently, an increase in the abdominal aorta diameter. Second, the progression of aortic dissection to aortic aneurysm might have resulted in acute compression of the duodenum. Cambria et al. reported that the combination of an aneurysm and aortic dissection increased the risk of rupture, especially in aortic dissection cases developing into aneurysms [7]. Despite liberal antihypertensive therapy under intensive care management for spinal cord ischemia, the aneurysm expanded within a short period.

The following factors other than AAA expansion could have developed SMA syndrome. First, spinal cord ischemia resulted in intestinal paralysis. Because spinal cord ischemia could have occurred up to the level supplied by arteries from the false lumen in the descending and abdominal aorta, the involvement of the lower thoracic spinal cord could have contributed to the bowel dysfunction. In traumatic spinal cord injury, the severity, level, and duration of spinal cord injury are associated with severe gastrointestinal dysfunction [8]. In our case, intestinal paralysis due to spinal cord ischemia may not have had a significant impact on the development of SMA syndrome because spinal cord ischemia produced incomplete paralysis and the injury level was below the thoracic spinal cord. Second, bed rest and fasting similarly impaired intestinal motility. Third, proximal dilation could be secondary to duodenal edema caused by these factors and worsened the obstruction in a vicious cycle. Although we could not distinguish these pathophysiologies, the complication of aortic dissection could have been directly and indirectly related to the development of SMA syndrome.

To our knowledge, this is the first case report of aneurysmal SMA syndrome with acute aortic dissection that developed within a short period in the acute phase. The patient had no significant gastrointestinal symptoms prior to the onset of acute aortic dissection. The initial CT image showed no signs of duodenal obstruction on admission. When the patient was diagnosed with SMA syndrome, the duodenum was drastically compressed between SMA and aortic aneurysm to the extent where it was hardly visible on the CT. Generally, SMA syndrome gradually develops because intra-abdominal fat gradually decreases. However, SMA syndrome associated with aortic aneurysm suddenly developed in this patient due to the rapid aneurysm expansion, which differed from the SMA syndrome caused by weight loss.

There is no established treatment for aneurysmal SMA syndrome. In our case, a thoracoabdominal aortic replacement was performed because both the AAA and acute aortic dissection were indications for surgical or endovascular repair. The patient had a surgical risk of malnutrition due to the SMA syndrome and spinal cord ischemia exacerbation. Therefore, the indication for highly invasive surgery was carefully determined in this case. Endovascular treatment could not decompress the duodenum early and directly. Moreover, stent grafting of the descending aorta combined with open surgery for the abdominal aorta may result in spinal cord ischemia progression. Because the patient had complicated type B aortic dissection for which early invasive treatment was appropriate, staged surgery with the development of collateral blood flow to the spinal cord was not indicated. Considering the benefits of early enteral nutrition and the risk of spinal cord ischemia progression, we chose to perform a thoracoabdominal aortic replacement. Fortunately, the postoperative course was uneventful, and the gastrointestinal symptoms improved. This report suggests that surgery is effective for aneurysmal SMA syndrome, even in high-risk patients, and an inability to orally consume nutrients is an indication for surgery.

## Conclusions

Clinicians should consider gastrointestinal symptoms in AAA as warning signs of SMA syndrome due to aortic aneurysm expansion, which can progress in a short time with accompanying acute aortic dissection. The complication of aortic dissection could have led to acute-onset SMA syndrome with the expansion of the AAA and other factors potentially associated with the clinical course of the disease.
